# EEG Alpha Band Synchrony Predicts Cognitive and Motor Performance in Patients with Ischemic Stroke

**DOI:** 10.3233/BEN-2012-129007

**Published:** 2013

**Authors:** Sviatlana Dubovik, Radek Ptak, Tatiana Aboulafia, Cécile Magnin, Nicole Gillabert, Lara Allet, Jean-Michel Pignat, Armin Schnider, Adrian G. Guggisberg

**Affiliations:** ^1^Division of NeurorehabilitationDepartment of Clinical NeurosciencesUniversity Hospital of GenevaGenevaSwitzerland; ^2^Division of NeurologyDepartment of Clinical NeurosciencesUniversity Hospital of GenevaGenevaSwitzerland

**Keywords:** Electroencephalography, stroke, functional networks, alpha band, plasticity

## Abstract

Functional brain networks are known to be affected by focal brain lesions. However, the clinical relevance of these changes remains unclear. This study assesses resting-state functional connectivity (FC) with electroencephalography (EEG) and relates observed topography of FC to cognitive and motor deficits in patients three months after ischemic stroke. Twenty patients (mean age 61.3 years, range 37–80, 9 females) and nineteen age-matched healthy participants (mean age 66.7 years, range 36–88, 13 females) underwent a ten-minute EEG-resting state examination. The neural oscillations at each grey matter voxel were reconstructed using an adaptive spatial filter and imaginary component of coherence (IC) was calculated as an index of FC. Maps representing mean connectivity value at each voxel were correlated with the clinical data. Compared to healthy controls, alpha band IC of stroke patients was locally reduced in brain regions critical to observed behavioral deficits. A voxel-wise Pearson correlation of clinical performances with FC yielded maps of the neural structures implicated in motor, language, and executive function. This correlation was again specific to alpha band coherence. Ischemic lesions decrease the synchrony of alpha band oscillations between affected brain regions and the rest of the brain. This decrease is linearly related to cognitive and motor deficits observed in the patients.

## 1. Introduction

Brain at rest displays synchronous spontaneous fluctuations [1]. These fluctuations are highly organized and coherent within neuro-anatomical and functional systems previously identified in task-induced activation studies. Hence, the analysis of functional connectivity (FC) at rest can deliver important information on neuronal organization and functioning. The major advantage of this approach is taking into account the network character of brain functioning and the possibility to analyze the whole brain with a single recording.

FC can be also assessed by means of electroencephalography (EEG) and magnetoencephalography (MEG). In contrast to fMRI these methods are rich on data with high temporal resolution and may reveal additional information on brain organization. Studies using EEG/MEG data for FC analysis reported that it is possible to localize the cortical generators of cortico-muscular interaction [2,3] and to reconstruct cortical network interactions [4]. Studies in tumor patients could identify a significant decrease in dysfunctional parts of the brain affected by the tumor [5,6]. These observations suggested that the magnitude of interregional MEG/EEG FC might be related to function.

ISSN 0953-4180/13/$27.50 © 2013 – IOS Press and the authors. All rights reserved

In this study we aimed to understand how brain lesions influence synchronous electrical oscillations in resting-state networks and how these changes translate into neurological deficits in a population with subacute, unilateral, territorial ischemic stroke.

## 2. Methods

Twenty stroke patients [mean age 61 years, 9 females, mean NIHSS (National Institute of Health Stroke Scale) 13] and nineteen healthy participants (mean age 67 years, 13 females) participated in this study. The inclusion criteria for the patients were first-ever unilateral, ischemic stroke in the territories of the middle and/or anterior cerebral artery. Electrophysiological and behavioral data were collected three months post-stroke onset. EEG was recorded with a 128-channel EEG-system in awake, resting condition with eyes closed. Five minutes of artifact-free data were recalculated against the average reference.

Motor function was evaluated by means of the Jamar dynamometer, the Nine Hole Peg test, the Stroke Rehabilitation Assessment of Movement (STREAM) and the Fugl-Meyer score. They were averaged to a composite motor score (CMS).

Attention and executive function were assessed with verbal phonetic fluency (VF), verbal working memory (VWM), and spatial working memory tests (SWM) [7].

The electromagnetic neural activity at each grey matter voxel was reconstructed with an adaptive spatial filter ("beamformer") from the signal recorded by the entire EEG electrode array. FC maps of each patient were normalized to the mean IC value across all voxels by calculating z-scores in the canonical MNI brain space. Mean FC at each voxel of each patient was compared voxel-wise against the mean of an age matched healthy control population with $t$-tests for one sample, corrected for testing multiple voxels with a 1% false discovery rate. To further assert functional and topographical specificity of the correlations, we extracted mean alpha band FC values of 14 anatomical regions of interest (ROIs) using Anatomical Automatic Labeling (AAL) templates [8] and correlated ROI FC values with clinical scores.

## 3. Results

IC between electrodes over the affected hemisphere of stroke patients and all other electrodes was significantly reduced in the alpha frequency band.

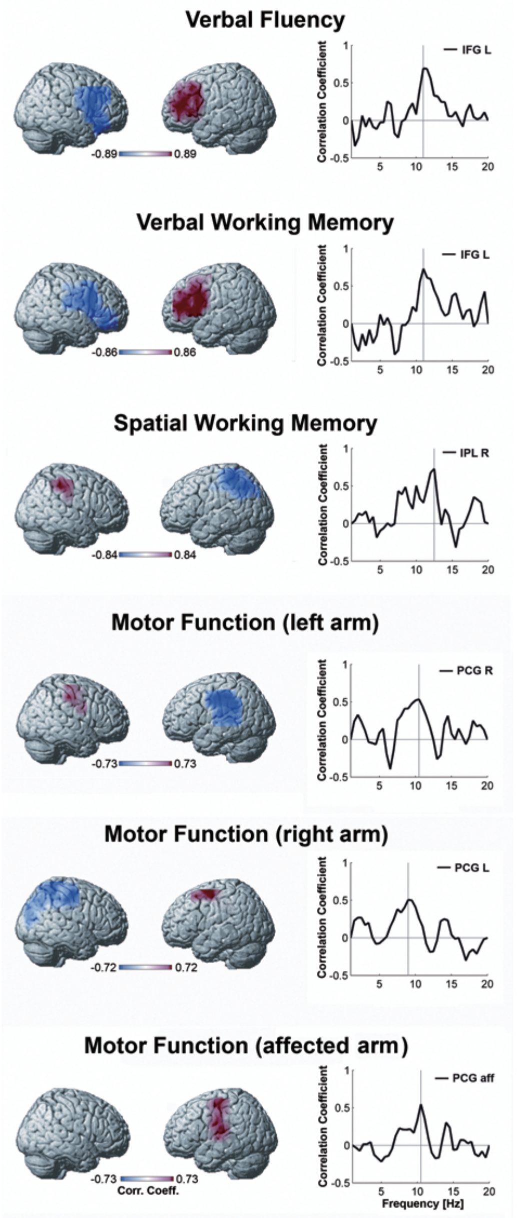

Fig. 1. Voxel-wise linear correlation of resting-state functional connectivity of the patients with their performance in clinical tests. Significant positive correlations (cluster corrected $p < 0.05$) could be observed only in nodes known to be involved in the processing of the tested function (e.g., in the left inferior frontal gyrus around Broca's area for verbal fluency and verbal working memory). These positive correlations were unique to the alpha frequency band (right column). Conversely, significant negative correlations with alpha band synchrony were found in the contralateral homologous counterparts (e.g., the right inferior frontal gyrus for verbal fluency/verbal working memory). For correlations with the composite motor score, the maps of patients with right hemispheric lesions were flipped in order to align affected and unaffected sides, in addition to a separate analysis of right and left sided motor function of all patients.

Local IC decreases in individual patients overlapped with their anatomical lesions, and corresponded exactly to the functional nodes that are known to be involved in the affected function. In addition, we observed increases of FC in non-lesion areas. These changes concerned most prominently the alpha frequency band.

Figure 1 illustrates the Pearson correlation analysis in the patients between the clinical performance after stroke and the mean resting-state FC at each voxel. Good performance in the VF and VWM tasks correlated with higher FC between the left fronto-opercular cortex and the rest of the brain. Similarly, the CMS correlated with FC in the contralateral motor cortex, and SWM performance with FC in the right inferior parietal cortex. Correlations between alpha FC and behavior therefore yielded maps of the function of the entire cortex. Moreover, significant negative correlations were observed between behavioral scores and FC in brain regions contralateral to the positive correlations. All these correlations were specific to FC in the alpha frequency band, and could not be observed in other oscillation frequencies. They remained significant and essentially unchanged in topography and frequency distribution when adjusting for age and lesion size with partial correlations.

The analysis of FC in anatomical regions of interest (ROIs) confirmed the functional specificity of the observed correlations. Alpha band FC between each ROI and the rest of the brain correlated only with functionally related, but not with unrelated clinical scores.

## 4. Discussion

This study reveals the electrical resting-state correlates of neurological deficits. It demonstrates that a disruption of coherent electrical oscillations at rest is linearly correlated with neurological deficits. A stroke-induced decrease in alpha band coherence between a given node and the rest of the brain is highly predictive of deficits in the function of the node, independent of anatomical lesions in this area. FC in dysfunctional brain areas was reduced most consistently in the alpha frequency band, and alpha band FC was a unique correlate of cognitive and motor function. Hence, alpha synchrony is a specific electrical biomarker of neurological function in patients with brain lesions.

The opposite correlations observed between alpha band FC and clinical data among homologous areas of both hemispheres have not been reported in fMRI studies. These negative correlations are specific to the alpha frequency and may correspond to a hyperactivity of the unaffected hemisphere of stroke patients which results from reduced inhibitory influences from the affected hemisphere. This misbalance in interhemispheric inhibition is well-known from functional imaging [9] and TMS studies [10]. Our results suggest that contralesional disinhibition is associated with a relative hypersynchrony in alpha oscillations.

The ability of a single resting-state EEG recording to capture the network basis of cognitive and motor deficits in patients offers new perspectives for research and clinical practice. The low costs and ubiquitous availability of EEG, the simplicity of handling, and the excellent sensitivity and specificity of the measure make this approach highly suitable for applications in therapy and rehabilitation.
